# Sickle cell disease chronic joint pain: Clinical assessment based on maladaptive central nervous system plasticity

**DOI:** 10.3389/fmed.2022.679053

**Published:** 2022-09-20

**Authors:** Tiago da Silva Lopes, Samir K. Ballas, Jamille Evelyn Rodrigues Souza Santana, Pedro de Melo-Carneiro, Lilian Becerra de Oliveira, Katia Nunes Sá, Larissa Conceição Dias Lopes, Wellington dos Santos Silva, Rita Lucena, Abrahão Fontes Baptista

**Affiliations:** ^1^Graduate Program in Medicine and Health, Federal University of Bahia, Salvador, BA, Brazil; ^2^Adventist Neuromodulation and Neuroscience Laboratory, Bahia Adventist College, Cachoeira, Brazil; ^3^NAPEN Network (Nucleus of Assistance, Research, and Teaching in Neuromodulation), São Paulo, SP, Brazil; ^4^Department of Medicine, Jefferson Medical College, Cardeza Foundation for Hematologic Research, Thomas Jefferson University, Philadelphia, PA, United States; ^5^Center for Mathematics, Computation, and Cognition, Federal University of ABC, São Bernardo do Campo, SP, Brazil; ^6^Graduate program of Medicine and Human Health, Bahiana School of Medicine and Public Health, Salvador, BA, Brazil; ^7^Department of Physiotherapy, General Hospital of the State of Bahia, Salvador, BA, Brazil; ^8^Laboratory of Medical Investigations 54 (LIM-54), Universidade de São Paulo, São Paulo, SP, Brazil

**Keywords:** musculoskeletal pain, symptoms assessment, red cell disorders, practical reasoning, evidence-based medicine

## Abstract

Chronic joint pain (CJP) is among the significant musculoskeletal comorbidities in sickle cell disease (SCD) individuals. However, many healthcare professionals have difficulties in understanding and evaluating it. In addition, most musculoskeletal evaluation procedures do not consider central nervous system (CNS) plasticity associated with CJP, which is frequently maladaptive. This review study highlights the potential mechanisms of CNS maladaptive plasticity related to CJP in SCD and proposes reliable instruments and methods for musculoskeletal assessment adapted to those patients. A review was carried out in the PubMed and SciELO databases, searching for information that could help in the understanding of the mechanisms of CNS maladaptive plasticity related to pain in SCD and that presented assessment instruments/methods that could be used in the clinical setting by healthcare professionals who manage chronic pain in SCD individuals. Some maladaptive CNS plasticity mechanisms seem important in CJP, including the impairment of pain endogenous control systems, central sensitization, motor cortex reorganization, motor control modification, and arthrogenic muscle inhibition. Understanding the link between maladaptive CNS plasticity and CJP mechanisms and its assessment through accurate instruments and methods may help healthcare professionals to increase the quality of treatment offered to SCD patients.

## Introduction

Sickle cell disease (SCD) is a set of hereditary diseases caused by substituting glutamine acid for valine at the sixth position of the hemoglobin β chains, which leads to the presence of hemoglobin S (HbS). Conditions such as low oxygen concentration, hypovolemia, and others can precipitate the structure twisting of HbS molecules fibers forming the sickle-shaped red blood cell membrane causing vaso-occlusive crises, which are the main reason for pain complaints in this population throughout life (1). The pain in SCD individuals can be acute or chronic and can emerge from nociceptive, inflammatory, and neuropathic mechanisms (2). SCD pain syndromes are classified as intermittent, persistent pain between vaso-occlusive crises and chronic pain complications (3).

Among the chronic pain complications, chronic joint pain (CJP) is a common condition in SCD that may also be associated with several musculoskeletal problems such as osteomyelitis, dactylitis, arthritis, and osteonecrosis both in adult and pediatric individuals (4–6). These chronic pain complications have a higher incidence in SCD and play an additional role in chronic pain generation (4, 5). The CJP may be focal when involving a single joint or multifocal when involving more than one joint (7). However, to date, few studies demonstrate the influence of maladaptive plasticity in the central nervous system (CNS) in the maintenance of CJP in SCD individuals, although these individuals have chronic pain with nociceptive, neuropathic, and possible nociplastic pain characteristics (8, 9). The presence of central sensitization, for example, is related to more episodes of pain crisis and frequent hospitalizations (10). Of utmost importance, few studies were developed explicitly for CJP in SCD.

The poor correlation between structural lesions, the intensity of self-reported pain (11), and the diffuse nature of the symptoms make CJP assessment a challenge for clinicians and healthcare professionals. In general, healthcare professionals have poor knowledge about pain neuroscience mechanisms (12) and reliable ways of assessing it (13, 14). This poor knowledge goes against the International Association Study of Pain (IASP) recommendation in the declaration of the Montreal meeting, which highlights that all people with pain have the right to have access to appropriate assessment and treatment of the pain by adequately trained healthcare professionals (15). Thus, considering the potential relation between CJP and central maladaptive plasticity in SCD individuals and the deficit in healthcare professionals' knowledge about pain neuroscience mechanisms and pain assessment, this review aims to highlight the mechanisms of CNS maladaptive plasticity that might be related to CJP in SCD and propose a battery for reliable musculoskeletal assessment adapted to those patients.

## Method

This review was carried out in the PubMed and SciELO databases, searching for information that could help in the understanding of the mechanisms of CNS maladaptive plasticity related to pain in SCD and that presented assessment instruments/methods that could be used in the clinical setting by healthcare professionals who manage chronic pain in SCD individuals. There was no limit placed on the publication year, and the searching was carried out through a combination of keywords such as Sickle Cell Disease and Joint Pain or Chronic Pain or Pain Assessment or Central Sensitization Evaluation or Painful Movement Assessment, Chronic Joint Pain and Cortical Reorganization or Arthrogenic Muscle Inhibition or Chronic Inventory Central Sensitization or Quantitative Sensory Test or Clinical Evaluation. In addition, the reference list of papers also was searched.

## Chronic joint pain in SCD: An overview of the problem

International Association Study of Pain defines pain as “An unpleasant sensory and emotional experience, associated with, or resembling that associated with, actual or tissue damage” (16). Pain plays a vital role in the organism's defense reaction to a hostile environment, and evidence of this is that in individuals with pain insensitivity, injuries are not perceived as such, decreasing life expectancy (17). On the other hand, chronic pain is persistent beyond 3–6 months, has no functional role, and is responsible for rendering dysfunctional several biological systems (18). In SCD, the constant joint tissue injuries secondary to the vaso-occlusive crisis are critical in developing chronic joint pain.

Primary afferent nociceptors richly innervate the joint in their capsule and synovium (19). These fibers are mostly from types Aδ and C and can be classified into two types: (a) True nociceptors; (b) Silent nociceptors. True nociceptors respond to mechanical nociceptive stimuli even in non-pathological conditions. As for the silent nociceptors, to respond to this type of stimuli, they must be primarily sensitized by inflammation-inducing aggressors (19, 20). The primary afferent nociceptors have on their membranes a wide variety of transient receptor potential ion channels that are responsible for the transduction of a wide variety of noxious stimuli arising from high magnitude mechanical, thermic, or chemical origins (21, 22). The nervous system sensitization occurs basically by neurogenic inflammation, mast cell activation, N-methyl-D-aspartate (NMDA) receptors activation, and glial activation (1, 2), which play an important role in the maintenance and subsequent pain chronicity in SCD individuals.

After joint tissue injury, the pro-inflammatory mediators such as bradykinins and prostaglandins interact with receptors or transient receptor potential vanilloid type 1 (TRPV1) of nociceptive fibers and sensibilize them to augment their response to a noxious stimulus (2, 22). Once activated, the nociceptors release peptides and neurotransmitters such as calcitonin gene-related peptide and substance P, which further contribute to the inflammatory response, causing vasodilation, swelling, and mast cell activation. Mast cells act by degranulation of histamine, which further sensibilize nociceptors (23). Interestingly, serum levels of substance P are increased in SCD individuals during the vaso-occlusive crisis and baseline state (24) and have been associated with to use of hydroxyurea (25). This cascade of biochemical events lowers the activation threshold of true nociceptors and recruits previously unresponsive silent nociceptors, which induce hyperalgesia and allodynia in joint pathologies in SCD individuals (26).

N-methyl-D-aspartate receptors are involved in the long-term potentiation process and are a crucial player in the chronicity of pain (27). At the spinal cord level, the constant nociceptive information arrives in the dorsal horn and provoke the release of glutamate neurotransmitter in presynaptic terminals that interacts with NMDA receptors post-synaptic (3). When NMDA receptors are activated, the nitric oxide is synthesized in the presynaptic terminals, increasing the expression of voltage-gated Ca2+ channels mainly responsive to P substance and glutamate (3). Concurrently, glial activation releases pro-inflammatory cytokines and more glutamate in this synaptic environment (1, 3). Thus, these series of intracellular signaling cascades augment and facilitate the transmission of nociceptive information.

**Figure 1:**
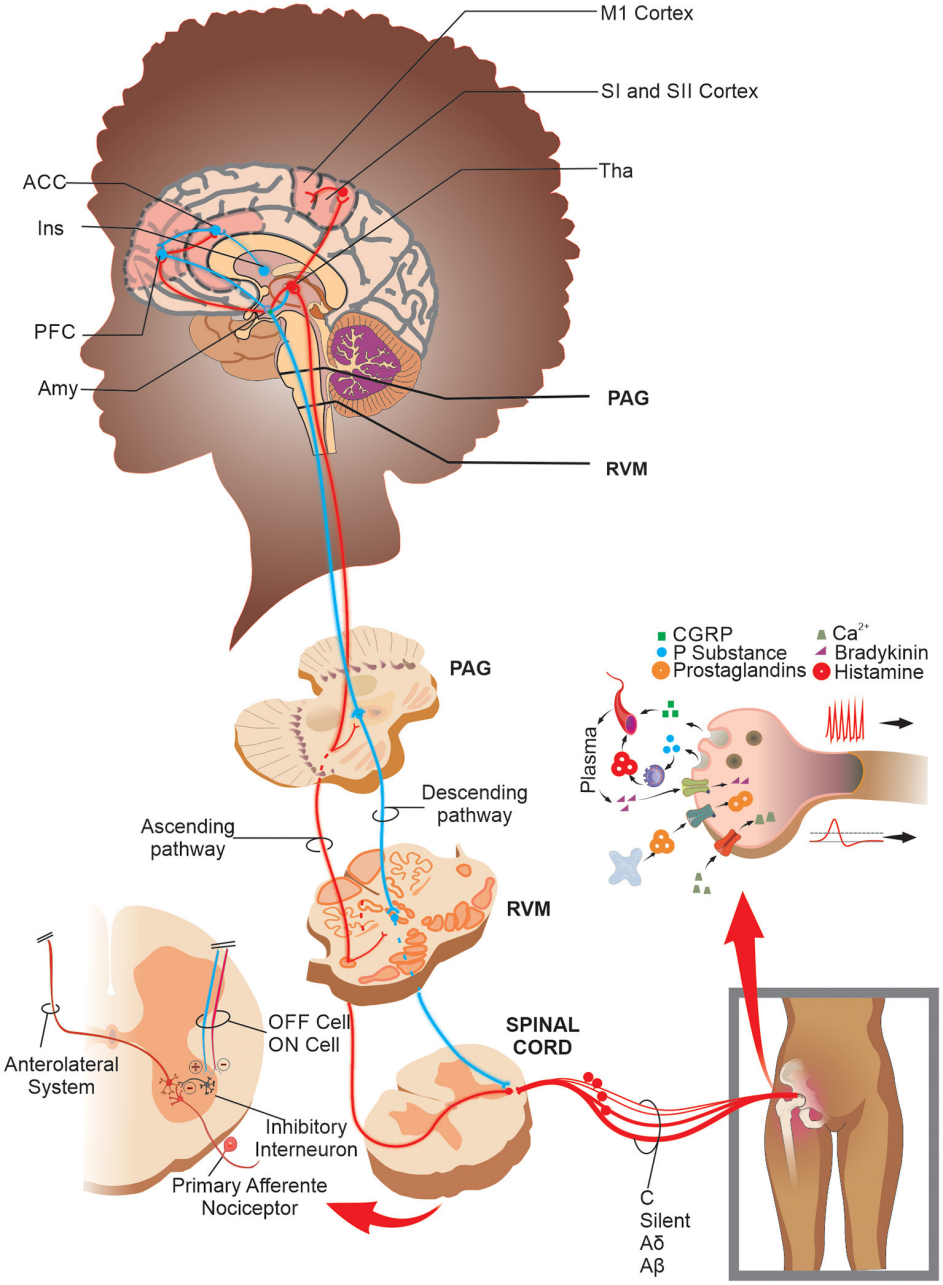
Schematic drawing of the peripheral sensitization, processing, and nociceptive modulation in joint pain: After a noxious stimulus in the joint, the TRP channels in true nociceptors transduce the nociceptive information and lead it to second-order neurons in the spinal cord. In addition, when there is a joint injury, a massive release of the pronociceptive chemical substances in/by free nerve endings promotes a depolarization threshold decrease and an increase in firing frequency rate in both true and silent nociceptors and mechanical receptors. The nociceptive information reaches the CNS, which processes and modulates it through brain networks and the PAG-RVM system. Specifically, in SCD patients, there is increased functional connectivity in areas such as PFC, ACC, M1, SI, and SII cortices. Abbreviations: ACC, anterior cingulate cortex; Amy, amygdala; CGRP, calcitonin gene-related peptide; Ins, insula; M1, primary motor cortex; PAG, periaqueductal gray matter; PFC, prefrontal cortex; RVM, rostral ventromedial medulla; SI, primary somatosensory cortex; SII, secondary somatosensory cortex; Tha, thalamus; TRP, transient receptor potential.

These nociceptive information reaches higher encephalic areas, such as Rostral Ventromedial Medulla (RVM), Periaqueductal Gray Matter (PAG), thalamus, amygdala, anterior cingulate cortex, somatosensory, prefrontal, and motor cortices (28, 29) that process and modulates the nociceptive information. However, nociceptive modulation can occur before reaching the thalamus and other brain structures (30). Once that nociceptive information reaches the thalamus, it processes it and redirects it to cortical areas of the primary and secondary somatosensory cortex through thalamocortical and thalamus-amygdala connections (29). The PAG, in turn, receives inputs from these superior centers and sends them to the RVM medulla, which through axonal fibers of “on” and “off” cells, modulate neuronal activity, facilitating or inhibiting the transit of nociceptive information in the dorsal horn of the spinal cord both presynaptic and post-synaptic (30, 31). This complex endogenous mechanism forms a pain processing and control system, often presenting a maladaptive function in chronic joint pain (Figure 1).

## Maladaptive CNS plasticity mechanisms and ways to evaluate it

### Dysfunction of descending inhibitory control in CJP

Central nervous system has various ways of inhibiting the input of pain information to higher processing centers. Descending inhibitory control is a mechanism of diffuse pain inhibition. Studies with conditions of CJP similar to SCD, such as hip and knee osteoarthritis, showed that the descending inhibitory control dysfunction might be an important triggering factor for central sensitization and chronic pain (32). Although some studies have found no consistent results about dysfunctions of descending inhibitory control in adult SCD individuals (10, 33), neuroimage data from another study with adult SCD individuals showed that there is an increased resting-state functional connectivity between the PAG and cerebellum in SCD individuals (34) which can affect RVM's “on” and “off” cells activity. In pediatric SCD individuals, the dysfunctions of the descending inhibitory control are few explored, but data from non-SCD individuals has shown that deficient endogenous pain inhibition can stem from painful experiences during infancy (35, 36). Therefore, these data make us think that the function of the descending inhibitory control system in SCD still needs to be better understood and evaluated in the clinical context.

**Figure 2:**
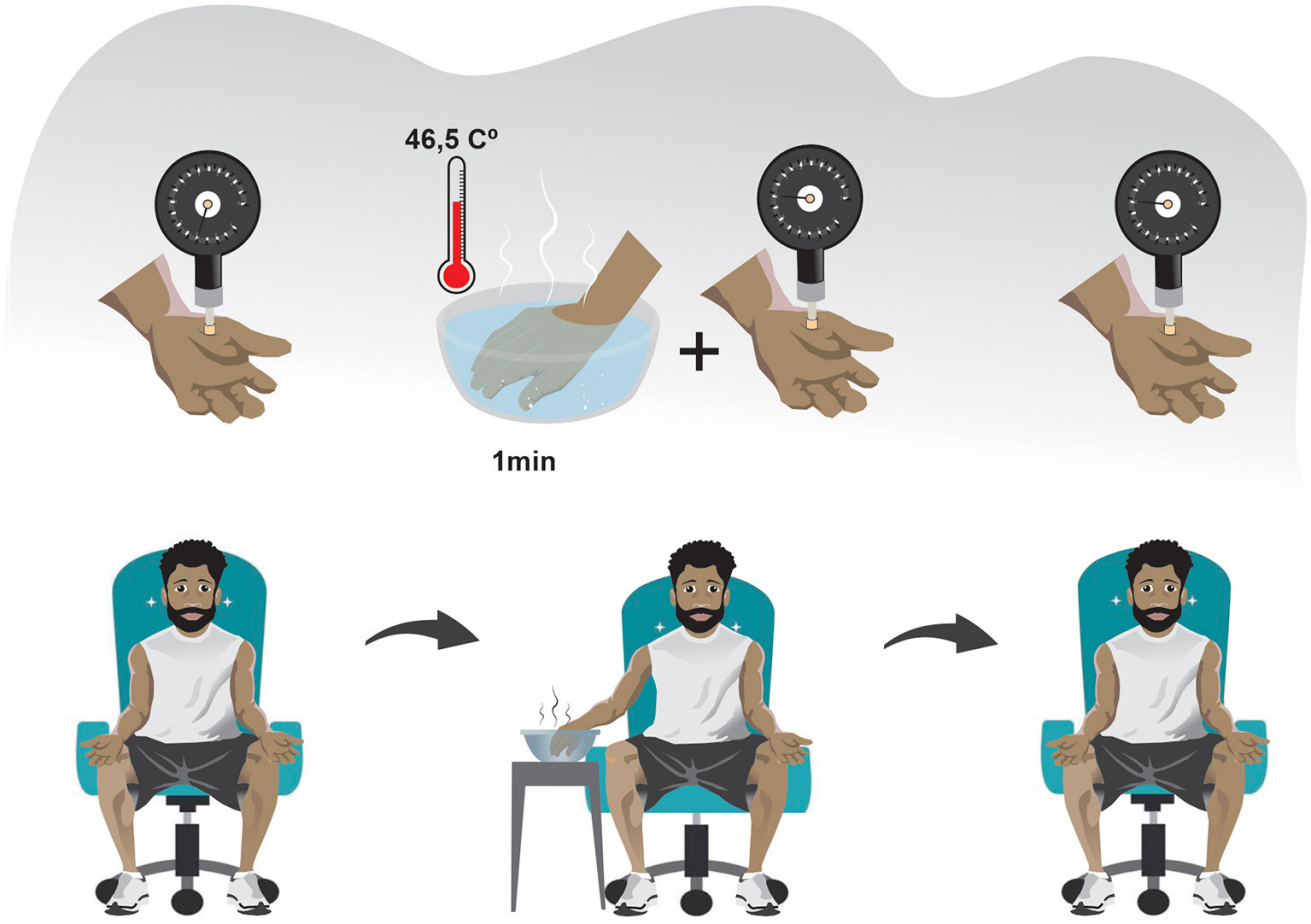
Schematic drawing of the conditioned pain modulation assessment: CPM can be assessed in three steps in the clinical setting. (1) A PST is made in the non-dominant side of the body (usually the thenar eminence). (2) After a sufficient period so that the pain caused by the PST has ceased, a PCST is made in a heterotopic region distant from the initially stimulated region and preferably on the contralateral side of the body, lasting 1 minute. (3) The PST is again applied immediately after or concomitantly to a PCST. Abbreviations: CPM, conditioned pain modulation; PST, painful stimulus test; PCST, painful conditioning stimulus test.

One of the traditional ways of assessing descending pain inhibitory system is through the paradigm of Conditioned Pain Modulation (CPM), previously known as “counter-irritation,” “pain inhibits pain,” and “heterotopic noxious conditioning modulation,” and “diffuse noxious inhibitory control”(37). This phenomenon is activated after a set of intense and/or noxious stimuli, making it a protective endogenous response to aggression. The evaluation of descending inhibitory control by the CPM method should be recommended for SCD individuals due to the malfunctioning of this mechanism is closely related to the persistence of joint pain in musculoskeletal conditions such as osteoarthritis and temporomandibular dysfunction (32, 38, 39) (Figure 2). However, clinicians should be aware that the long-term pain, and the use of opioid agents can result in a reduced response of CPM scores (40).

In CPM assessment, a pressure threshold meter for applying painful mechanical stimulus in the thenar region of the non-dominant hand has a good coefficient of intra-session reliability (ICC >0.75). It seems to be a reliable method for performing a Painful Stimulus Test (PST). The pain caused by the mechanical stimulus must be of moderate intensity (41, 42). In turn, the Painful Conditioning Stimulus Test (PCST) can be done with cold or hot water. However, the immersion of the dominant hand in a water vessel with a temperature of 46.5°C has a good Intraclass Correlation Coefficient (ICC = 0.79) (42) and is more recommended in the SCD context because it can avoid a vaso-occlusive crisis during the evaluation. Using a thermometer to verify the heat dissipation and ensure the ideal temperature during immersion and using the same kilograms-force generated by the pressure threshold meter during the PST before and after the PCST may decrease potential measurement biases (41, 42).

The quantification of the CPM can be made according to the following equation:


$$\begin{array}{l} CPM=piPST1-piPST2 \end{array}$$

Where piPST1 corresponds to the pain intensity caused by the first painful stimulus test and piPST2 pain intensity caused by a second painful stimulus test. A positive result indicates the presence of a preserved descending inhibitory control, while a negative result indicates the opposite (42).

### Central sensitization in CJP

Previously, the term “centralized pain” was often used to classify the pain experienced by patients with central sensitization. However, this term was not part of recognized by the IASP. Following the proposition of a research group (43), an IASP force task recently added a new pain term called “nociplastic pain” into the list of taxonomic definitions for pain (44), even though it caused a comprehensive discussion related to its real need and the best way for it to be defined (45–47). This new term proposes to differentiate the “pain that arises from altered nociception, despite there is no clear evidence of actual or threatened tissue damage that causes peripheral nociceptor activation or evidence of disease or injury to the somatosensory system that causes pain” from those kinds of pains typically classified only as nociceptive or neuropathic.

The central sensitization mechanisms involve the perpetuation of joint pain that can be favored by poor descending inhibitory control, which over time causes phenotypic alteration of Aβ fibers specialized in conducting non-painful stimuli (30). In addition, nociceptive information is not properly inhibited in the dorsal horn of the spinal cord and advances freely until it reaches higher areas of the nervous system, causing a central sensitization of multiple structures (48). Central sensitization of multiple structures involves a maladaptive change of important anatomic/functional networks that process information in all pain dimensions, i.e., sensory, emotional, and cognitive (49). Due to the important role in pain processing, these anatomic/functional networks are called the pain connectome (49).

In conditions of chronic non-SCD pain, the default mode network (DMN), the salience network (SLN), the sensorimotor network (SMN), and the antinociceptive system are connectome strongly affected by central sensitization (49, 50). The DMN includes the medial prefrontal cortex, posterior cingulate cortex, precuneus, and lateral parietal cortices and is activated in a resting state of the mind when the individual is instructed not to think about anything specific (49). Next, SLN comprises the bilateral insula cortices, anterior cingulate cortex, and middle cingulate cortex and is activated by salient stimuli that stand out from the environment (e.g., nociceptive stimulation caused by the movement of an inflamed joint) (51). The SMN includes bilateral primary and secondary somatosensory cortices, primary motor (M1) cortex, and the supplementary motor area (SMA) and is involved in the descriptive sensory processing of pain (51). Finally, the antinociceptive system comprises the PAG and RVM, which, as previously discussed, are core structures involved in pain modulation (49). This pain connectome is dynamic due to the capacity to generate connections within and between themselves (49).

In SCD individuals with chronic pain, some studies using functional Magnetic Resonance Image (fMRI) alone or coupled with Electroencephalography (EEG) have found a maladaptive change in the pain connectome (34, 51–53). Their results showed that SCD individuals with high levels of pain and hospitalizations had an increased resting-state functional connectivity between SLN, DMN, and SMN structures (e.g., dorsal anterior cingulate cortex and the right precuneus, secondary somatosensory cortex, and the left precuneus, inferior parietal lobule and the middle cingulate cortex, right posterior cingulate cortex and the right primary somatosensory cortex) when compared with individuals with low levels of pain and hospitalizations (52). SCD individuals also presented hypoconnectivity of SMN structures (i.e., motor cortex) compared to healthy controls and between other regions outside of the SMN, such as the dorsolateral prefrontal and parietal cortices (51). In addition, this same study found that SCD increased functional connectivity between DMN and SLN structures (e.g., precuneus/ posterior cingulate cortex and temporal regions) (51). Finally, studies comparing SCD individuals and healthy controls found changes in functional connectivity of the PAG (a core structure of the antinociceptive system) (34, 53). Functional connectivity between the PAG and the anterior cingulate cortex (a structured core of SLN) is decreased in SCD patients when compared to healthy individuals but increased between the PAG and several cortical regions that play functions of sensory processing, motor processing/executive function, emotion and memory/learning when SCD patients were compared with those without pain (53).

The sensitization of the pain connectome may be associated with multiple musculoskeletal and non-musculoskeletal symptoms found in individuals with severe chronic pain. These include decreased pain threshold, expansion of pain receptive field to further regions unrelated to pain, interpretation of non-painful stimuli as painful, photophobia, bowel diseases, and sleep, attention, and mood-altering (54, 55). The emergence of these phenomena may trigger the change of the clinical status from a musculoskeletal disease to a multi-systems disease. Typically, those symptoms are under-evaluated by clinicians and are not related to the presence of persistent pain. However, these aspects are essential as they help in decision-making and prediction of patient outcomes, as evidenced by a study that showed that individuals with central sensitization due to chronic pain secondary to osteoarthritis of the knee are five times more likely to have pain refractory to surgical treatment of total knee arthroplasty (56). In SCD individuals, central sensitization has been associated with increased vaso-occlusive crises, poor sleep quality, and psychosocial disorders (10). For this reason, this should be considered during the evaluation since this is probably one of the main causes of refractory joint pain (57).

Sensitivity hyperphenomena, such as allodynia or hyperalgesia to thermic and vibratory stimuli, and mechanical and thermal temporal summation, have been associated with central sensitization in non-SCD individuals with chronic pain (57). These sensitivity deficits are also found in pediatric SCD individuals, among lower mechanic pain, cold pain, heat pain, thermal detection thresholds, and heat pain tolerance (58). In addition, studies with adult SCD individuals showed that they also present sensory alterations expressed by a higher intensity of cold pain, heat pain, thermal temporal summation, and mechanic pain is found in compared with healthy controls (33, 59).

Some methods are essential in evaluating central sensitization/nociplastic pain characteristics in clinical and research settings because they can help evaluate whether CJP in SCD is influenced and/or supported by central sensitization. The central sensitization inventory (CSI) is an evaluation instrument that, although non-specific to SCD, is highly recommended to be used in clinical practice in the SCD context (54). The CSI is divided into two parts, A and B. In part A, 25 descriptive alternatives of multidimensional symptoms are associated with central sensitization. Each alternative has a score varying from zero (never) to four (always), with a maximum total score of 100 points. In part B, 10 alternative clinical conditions are recognized as central sensitivity syndromes (CSS) (60). The cut-off at 40 points has excellent levels of sensitivity (81%), specificity (75%), positive predictive (2.93), and negative predictive value (0.52) to recognize central sensitization (60). However, despite these good diagnostic accuracy values, the CSI still needs to be validated in SCD individuals, and its results should be interpreted with caution. Due to the need for severity ratings of central sensitization, a 10-point classification with severity intervals was created, consisting of the following categories: subclinical (≤29), mild (30–39), moderate (40–49), severe (50–59) and extreme (≥60) (61). This severity rating allows better utilization of CSI in clinical practice and may help as a parameter of the therapeutic response. This instrument has been culturally translated and validated in several languages (62, 63).

Quantitative sensory tests (QST) are another way to assess central sensitization (10, 57, 64). All systematic sensory evaluations that allow quantified responses can be viewed as a QST. However, a set of QST (mechanical, thermal, and vibratory) was standardized to evaluate the integrity of the somatosensory system and to guarantee the accuracy and reproducibility of the findings (65). QST protocols consider several sensory parameters, as well as biological aspects ranging from body temperature to trophic changes in the musculature (64, 65). However, although QST protocols can be performed in both bedridden and non-bedridden individuals, their complete execution is time-consuming and can be impracticable in some clinical contexts. In this context, there are some attempts to validate a bedside QST as a low-cost and time-efficient alternative (66, 67).

The bedside QST can be easily applied in clinical routine, and its execution does not require a large training time. Studies showed that bedside QST protocol using low-cost equipment could be used in each step of the sensory assessment procedure, such as (a) 3 cm^2^ metal coin/piece with 22°C or 37°C (cold/warm detection thresholds); (b) cotton wool/Q-tip (mechanical detection threshold); (c) tuning fork (vibration detection threshold); (d) 10-ml syringe sealed or toothpick (mechanical and pressure pain threshold); (e) glass vial filled with hot water 40°C or metal pieces with 45°C (heat pain threshold); (f) ice cubes in a plastic bag or metal piece with 8°C (cold pain threshold); (g) toothpick (temporal summation) (66, 67). However, the correlation between bedside QST and standard QST protocol is variable and impacted by the expertise of a healthcare professional.

A study proposed three steps of a decision tree that helps clinicians to interpret the findings of QST evaluation of mechanical detection threshold (Aβ fibers), cold pain (Aδ fibers), and heat pain (C fibers), specifically in SCD individuals (8). In the clinical setting, QST stimuli should be evaluated in both painful and non-painful sites. In the first step, if all QST findings are negative, the clinical interpretation must be that there is no central or peripheral sensitization. In the second step, if mechanical stimuli findings in the non-painful site are positive, then the clinical interpretation must be that there is central sensitization. In the third step, if cold or heat pain is present in the painful site and these same painful stimuli result negative in the non-painful site, then the clinical interpretation must be that there is peripheral sensitization. Finally, the decision tree proposes that if all three steps result in negative findings, then the interpretation must be that there is mixed pain (8).

The safety of the QST protocol in the clinical setting has been previously tested in subjects with SCD, and there was no perpetuation or worsening of pain after its application (8). However, attention is necessary because data show that after QST testing in SCD patients, there are changes in pro-inflammatory biomarkers such as increased levels of Interleukin 6 (IL-6), substance P, and tumor necrosis Factor-alpha (TNFα) (33). In SCD, the thermal pain threshold (TPT) to cold <17.01°C and heat <43.91°C are indicative of impaired nerve sensitivity, and pressure pain threshold (PPT) <4.42 g is indicative of the existence of altered sensory function (68). Thermal pain threshold (TPT) assessment with temperature in 32°C baselines and an increasing/decreasing temperature at a rate of 1.5°C/s is used in clinical settings (ICC >0.55) (69). In cases of non-SCD pain, specifically osteoarthritis of the knee, the PPT increasing pressure at a rate of 0.5 kgf/s has a good diagnostic reliability value varying according to the evaluated joint site (ICC: 0.64–0.73) (70).

Finally, another way of assessing central sensitization in individuals with SCD uses its typical clinical criteria checklist (71) developed by a consensus of experts. Although this checklist is non-specific to SCD individuals, it is also useful for clinicians and healthcare professionals because it helps identify signs and symptoms characteristic of central sensitization, such as pain disproportionate to injury, disproportionate aggravating/easing factors, and psychosocial symptoms, and diffuse palpation. These discriminative items indicate the presence of central pain sensitization with excellent accuracy values (sensitivity 91.8%, specificity 97.7%, positive predictive value 91.8, and negative predictive value 97.7) (72). Thus, using these instruments during the evaluation of SCD individuals with CJP may help in the more precise knowledge of the mechanism underlying the patient's pain. This clinical criteria checklist provides a basis for better clinical decision-making and possibly less chance of non-adherence to the proposed treatment.

### Motor control modifications and cortical reorganization in CJP

In the face of pain, the neuromusculoskeletal system undergoes adaptive motor modifications that affect motor control and joint mechanics. These modifications have been studied over time due to the importance of their understanding for both clinicians and researchers. Therefore, one theoretical model (73) was established to clarify the interaction between pain and motor control changes making the following propositions: Firstly, the adaptation of the motor control to pain is a consequence of the redistribution of the activity within and between muscles. Secondly, the change in mechanical behavior initially has a protective function of preventing further pain or injury. However, in the long term, it involves changes in various levels of the nervous system, which lead to increased joint load, decreased mobility, and variability of movement and muscle weakness (73).

In the presence of CJP, motor and sensory primary cortical reorganization are associated with motor control impairment. This cortical reorganization has been demonstrated in non-SCD adult individuals with low back pain (74, 75), chronic lateral epicondylalgia (76), osteoarthritis of the knee (77), and chronic patellofemoral pain (78), but there is no study with pediatric individuals. This cortical reorganization is expressed through the overlap (i.e., blurring) or retraction in the areas of somatotopic representation of the motor homunculus. The greater the cortical reorganization, the greater the perpetuation of the pain (77).

The intracortical inhibitory system, modulated by tonic GABAergic activity, plays an important role in the development of cortical somatotopic representations. This specific function is due to mechanisms that differentiate cortical efferent motor actions, either by facilitating muscle activation during a motor task or by inhibiting undesirable muscular activations (79). Although changes in intracortical inhibition are not a consensus (80), intracortical inhibitory dysfunction mediated by GABAergic connections has been demonstrated in individuals with chronic pain (81) through Transcranial Magnetic Stimulation (TMS), a technique that has been often used to assess cortical connectivity.

**Figure 3:**
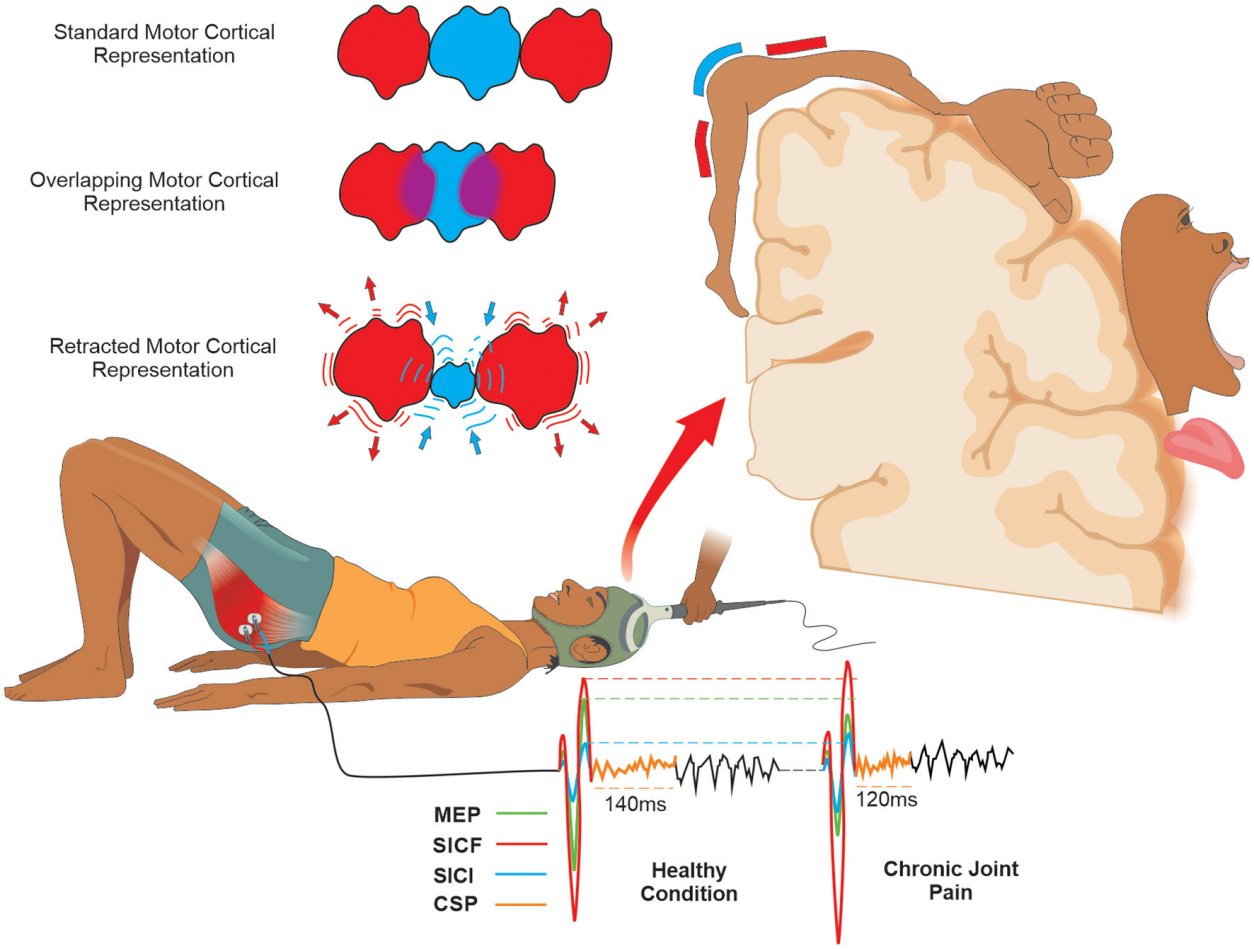
Schematic drawing of the measures of motor cortical excitability with tms and mapping of motor cortical representation in joint pain condition: Using TMS, Motor Cortical Excitability can be expressed by corticospinal (MEP) and intracortical measures (ICF, IIC, and ǂCSP). In the CJP, the CSP, SICI, and MEP are decreased, and the SICF is increased compared to the healthy condition. The Standard Motor Cortical Representation in the presence of pain can classically be changed into two possible basic forms. (1) An Overlapping of the Motor Cortical Representation adjacent to that affected by pain; (2) A Retraction of the Motor Cortical Representation affected by pain and an increase in the adjacent representation. ǂCSP is obtained only when the assessment is made during a muscle contraction. Abbreviations: CSP, cortical silent period; SICF, short-interval intracortical facilitation; SICI, short-interval intracortical inhibition; MEP, motor evoked potential; TMS, transcranial magnetic stimulation.

Transcranial magnetic stimulation also allows the evaluation of muscles' cortical representations through cortical mapping. Briefly, cortical mapping through TMS is made using a set of pulses with intensity fixed in accord with a percentage of the maximal stimulator output (82). This set of pulses should be applied at various scalp sites using a figure-of-eight coil and a spatial coordinate system referenced to the vertex (83), and the amplitude of MEPs evoked in contralateral muscles is measured (82). However, although the assessment of cortical mapping through TMS can be useful in clinical settings, there are no studies evaluating its diagnostic reliability (Figure 3).

In SCD individuals, CJP is possibly associated with maladaptive motor behavior and cortical representation changes due to their chronic and disabling pain (7). The changes in functional connectivity of the structures are involved in the descending inhibitory control of nociceptive information in individuals with SCD (34) and can be associated with intracortical inhibition (84). Thus, although TMS evaluation is not specific to SCD individuals, clinicians and healthcare professionals should be used to investigate these possible cortical alterations both in adult and pediatric SCD individuals with CJP.

### Arthrogenic muscle inhibition and CJP

It is common that after joint injuries, there is the presence of weakness in the adjacent involved musculature. The possible cause for this muscle weakness is the presence of a central reflex inhibition that can provoke a failure to fully recruit the motor units and/or a suboptimal firing of the motor units that are recruited (85), preventing the complete activation of the surrounding musculature to the injured joint during a maximal voluntary muscular contraction. This phenomenon has been called Arthrogenic Muscle Inhibition (AMI) (86).

Arthrogenic muscle inhibition can be interpreted as a mechanism of physiological protection to prevent new lesions and potentiation of tissue repair (87). However, AMI may persist for several months or even years after injury (88). This persistence may compromise the rehabilitation process by negatively impacting strengthening protocols, thus, contributing to injury progression and associated dysfunction (87). Common conditions such as joint pain, ligamentous laxity, and joint effusion are potential factors that facilitate the establishment of AMI (86).

In the AMI, there is an alteration of the firing of the joint receptors that send signals for the medullary inhibitory interneurons, causing inhibition of the activity of the alpha motoneurons and, consequently, the musculature involved in the affected joint (87). Joint pain may contribute to the AMI due to the alteration of the excitability of the flexor reflex pathway (86), which has the characteristic of facilitating the flexor and inhibiting the extensor muscles in the region surrounding the painful joint (89). In addition, joint pain in the knee has been associated with decreased muscle activation of the quadriceps (90, 91).

Although a systematic review has shown that the mechanisms of AMI are mostly studied in knee joint injuries (91), it may also be observed in individuals with pathologies in the hip. In this condition, AMI may be represented by a decrease in Gluteus maximus activation during extension activity in pronation (92). In this sense, as the most affected joint in SCD is the hip due to avascular osteonecrosis (7), the healthcare professional must be aware of the possibility of AMI playing an important role in this condition. However, many SCD individuals are likely quite physically deconditioned due to limited physical activity because of fatigue (93) or concerns about triggering vaso-occlusive crises after physical activity (94). This clinical characteristic in SCD individuals can make AMI assessment challenging because of the potential confounding biases related to physical deconditioning or structural musculoskeletal alterations, especially in bilateral affections. On the other hand, in unilateral affections, these confounding biases can be minimized by comparison with the unaffected side. To date, no studies have evaluated AMI mechanisms in both adults and pediatric SCD individuals, and in future studies, the impact of physical deconditioning on AMI assessment should be better clarified.

**Figure 4:**
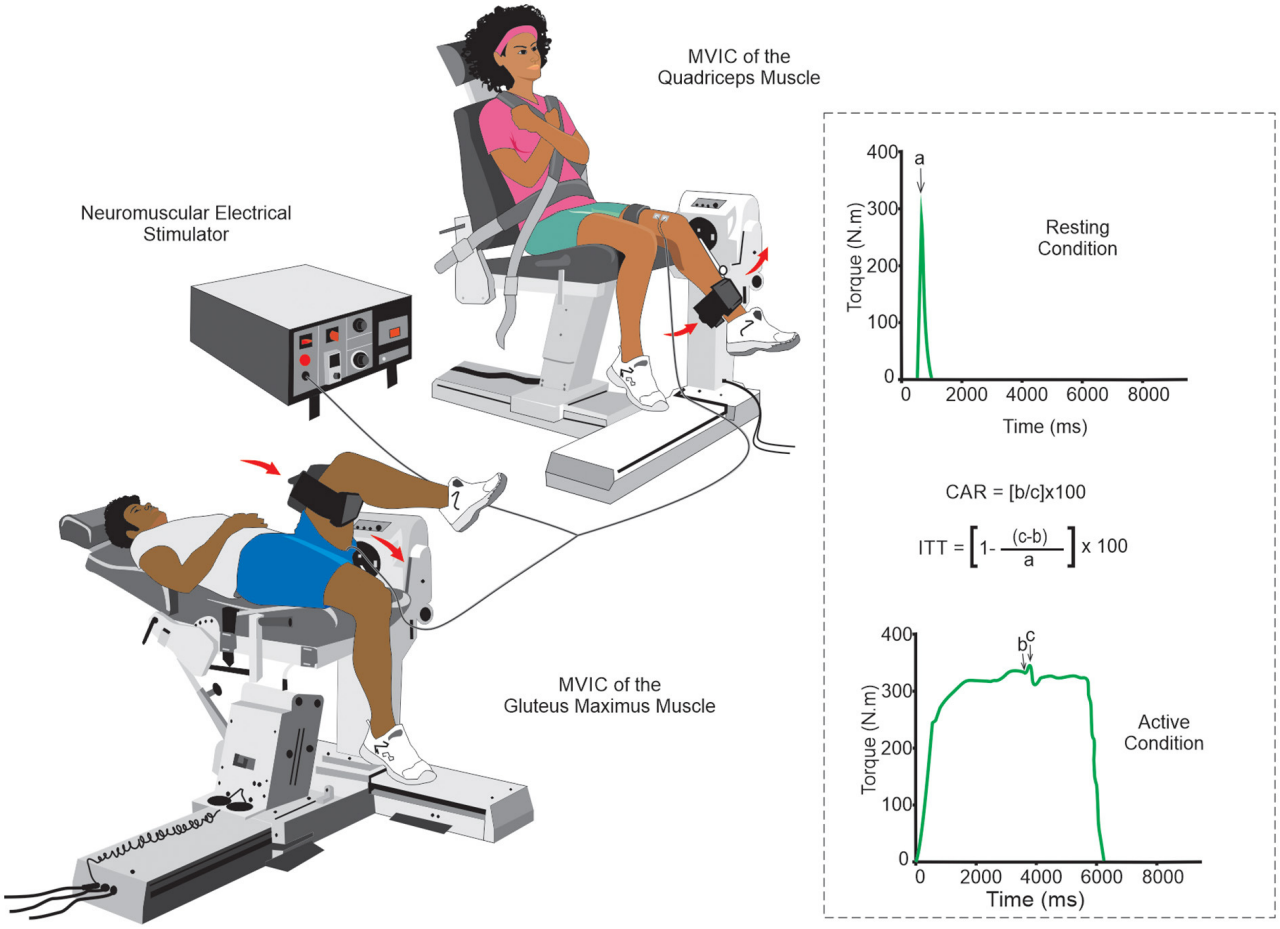
Schematic drawing of the arthrogenic muscle inhibition evaluation by central activation rate and interpolated twitch technique: Although the knee joint is the most evaluated with the CAR and the ITT methods, their methodological simplicity allows the assessment of AMI to be also used in other joints (e.g., the hip joint in the figure above). The quantification of the evaluation can be done using specific mathematical formulas for each method. a. stimulus-evoked torque at rest; b. voluntary torque at the time of stimulus delivery; c, peak torque evoked due to the electrical pulse. Abbreviations: AMI, arthrogenic muscle inhibition; CAR, central activation rate; ITT, interpolated twitch technique.

In the clinical setting, AMI can be assessed using two quantitative methods: the Central Activation Rate (CAR) and the Interpolated Twitch Technique (ITT) (85, 91, 95). In both methods, the individual is asked to make a maximal voluntary isometric contraction (MVIC), and the force/torque generated by the muscle is registered. Then, when the force/torque plateau is reached, a maximal or supramaximal electrical stimulus is introduced. However, in the ITT method, this electrical stimulus can also be made initially with the muscle at rest (85, 91, 95). For electrical stimulus, 10 pulses, 100 Hz,200 μs pulse duration, and 400 V appear to be a reliable stimulation parameters for muscle contraction (85). Individuals with CAR >95% have a muscle fully activated by voluntary central stimulation, and those with less than that have some central muscle inactivation (91). In the ITT method, the higher the index score, the greater the number of fibers that are not centrally activated (85). Both methods seem simple, easily performed, and therefore feasible in clinical practice and research (Figure 4).

When comparing CAR and ITT in the capacity to estimate the quadriceps muscle activation, there was a significant variation between methods, with an estimated difference of up to 5.5% (85). In addition, it is suggested that ITT is a more accurate measure since the CAR might overestimate voluntary muscle activation (85). Some articles have assessed and reported good reliability of these methods in knee joints (96, 97), and the CAR method was found reliable within- (ICC = 0.94) and between-measurement sessions (ICC = 0.86) (96) while in the ITT method the reliability within measures was (ICC = 0.89) (97). Unfortunately, no studies evaluated the diagnostic value of these methods in joints frequently affected by SCD individuals, such as the hip, shoulder, and elbow. Although both CAR and ITT are not specific to SCD individuals, these methods should be used in clinical practice to evaluate CJP in SCD individuals.

## Final remarks and conclusion

Chronic joint pain in patients with SCD might be related to maladaptive plasticity in the CNS, as it shares mechanisms with many known joint pathologies. Some of these maladaptive changes in the CNS are already known and include mainly poor descending inhibitory control, central sensitization, motor control impairments, reorganization of the motor cortex motor, and inhibition of induced maximal voluntary contraction. These changes may be assessed by a set of tests and/or questionnaires that are already available and could be useful in the clinical assessment and research in SCD. In the clinical setting, every healthcare professional can measure these maladaptive changes through instruments and methods with good diagnostic reliability (Table 1). These maladaptive plasticity changes may contribute to persistent pain in SCD, but there is a substantial lack of evidence regarding this aspect. However, future studies should be performed to elucidate and confirm these possible maladaptive changes in the nervous system in SCD individuals related to CJP to understand and treat the pain in those patients with better results.

**Table 1:** Summary of the main central nervous system maladaptive changes, assessment methods, clinical interpretation, and diagnostic reliability that can be used in CJP related to sickle cell disease.

**CNS maladaptive changes in chronic joint pain**	**Assessment methods or instruments**	**Clinical interpretation**	**Diagnostic reliability**
Insufficiency of descending inhibitory control	• Conditioned pain modulation (42)	• piPST1 > piPST2 = Descending inhibitory control is functioning •piPST1 < piPST2 = Descending inhibitory control system is faulty	• PST: Pressure threshold meter (ICC >0.75) •PCST: Hot water in 46.5°C (ICC = 0.79)
Central sensitization	• Central sensitization inventory (60,61)	• Severity ratings: Subclinical ( ≤ 29) Mild (30-39) Moderate (40-49) Severe (50-59) Extreme (≥60)	• Cut-off at 40 points: Sensitivity (81%) Specificity (75%) Positive predictive value (2.93) Negative predictive value (0.52)
	• Quantitative sensory test (68-70)	• TPT to cold <17.01°C and heat <43.91°C are indicative of impaired nerve sensitivity •PPT <4.42 g is indicative of the existence of an altered sensory function	• TPT: 32°C baselines with decreased/increased temperature at a rate of 1.5°C/s (ICC >0.55) •PPT: Increasing pressure at a rate of 0.5 kgf/s (ICC: 0.64–0.73)
	• Clinical criteria checklist (71,72)	• Pain disproportionate to injury disproportionate aggravating/easing factors; psychosocial symptoms; diffuse palpation	Sensitivity (91.8%) Specificity (97.7%) Positive predictive value (91.9) Negative predictive value (97.7)
Modifications of motor control and cortical reorganization	• Cortical mapping by transcranial magnetic stimulation (77)	• There is an overlap (blurring) or retraction in the areas of somatotopic representation of the motor cortex	
Arthrogenic muscle inhibition	• Central activation rate (91,96)	• When the central activation rate is below 95%, it is an indication that there are muscle fibers that are not being activated by central neural pathways	• CAR: Within- measurement (ICC = 0.94) Between-measurement (ICC = 0.86)
	• Interpolated Twitch Technique (85,87)	• The higher the proportion, the greater the number of muscle fibers that are not centrally activated	• ITT: Within measurement (ICC = 0.89)

Abbreviations: CAR, Central Activation Rate; ITT, Interpolated Twitch Technique; ICC, Intraclass Correlation Coefficient; PCST, Painful Conditioning Stimulus Test; piPST1, pain intensity of first Painful Stimulus Test; piPST2, pain intensity of second Painful Stimulus Test; PST, Painful Stimulus Test; TPT, Thermal Pain Threshold; PPT, Pressure Pain Threshold.

## Author contributions

All authors contributed to the development of the article in specific activities, such as planning, designing, and drafting/revising the final manuscript.

## Funding

This study was funded by the Research Support Foundation of the State of Bahia (FAPESB) through the Support Program for Emerging Center, 8133/2014 (Process: PNE0020/2014). TL is supported by a Ph.D. scholarship from FAPESB (Process: BOL1492/2018). AB receives a scholarship from Brazilian National Council for Scientific Development (CNPq) (Process: 305539/2021-3) and also supported by CEPID/BRAINN-The Brazilian Institute of Neuroscience and Neurotechnology (Process: 13/07559- 3). JS is supported by an MSc scholarship from the Coordination for the Improvement of Higher Education Personnel (CAPES).

## Conflict of interest

The authors declare that the research was conducted in the absence of any commercial or financial relationships that could be construed as a potential conflict of interest.

## Publisher's note

All claims expressed in this article are solely those of the authors and do not necessarily represent those of their affiliated organizations, or those of the publisher, the editors and the reviewers. Any product that may be evaluated in this article, or claim that may be made by its manufacturer, is not guaranteed or endorsed by the publisher.
